# Bioinformatic study to discover natural molecules with activity against COVID-19

**DOI:** 10.12688/f1000research.26731.1

**Published:** 2020-10-06

**Authors:** Sweta Singh, Hector Florez

**Affiliations:** 1 Savitribai Phule Pune University, Pune, India; 2 Universidad Distrital Francisco Jose de Caldas, Bogota, Colombia

**Keywords:** COVID-19, SARS-CoV-2, Molecular Docking, High throughput virtual screening

## Abstract

**Background: **In 2020, the world has struggled to deal with coronavirus disease 2019 (COVID-19), which started in 2019 in China and has spread throughout the globe, affecting at least 31,175,835 humans globally and claiming 962,634 lives reported till 22nd September, 2020 by the World Health Organization. The main causative agent for this disease is known as severe acute respiratory syndrome coronavirus 2 (SARS-COV-2). So far, there is no cure or proven therapeutics available till date. Therefore, we undertook this study to ﬁnd the most probable drug candidate through a bioinformatics study.

**Methods:** Thus, we virtually screened the Zinc natural database using HTVS tool through molecular docking studies to analyze molecules recommended for the treatment of COVID-19.

**Results: **Ramipril benzyl ester, propafenone dimer and Lariciresinol are three important drugs found from the present study due to their medicinal application which could be helpful in treating the disease. Stylopine, quillaic acid, cinobufagin, vitisinol C, segetalin A, scopolamine, 3-oxo glycyrrhetinic acid, conchinone B, lactimidomycin and cardinalins 4 are the other lead molecules that could be used as therapeutics against COVID-19 disease.

**Conclusions:** The studied molecules could act as an effective inhibitory drug against COVID-19.

## Introduction

A novel virus was first found infecting human in China on 30 ^th ^December, 2019. The causative agent for this disease was found to be severe acute respiratory syndrome coronavirus 2 (SARS-CoV-2). Coronavirus disease 2019 (COVID-19) was declared as a pandemic by the World Health Organization on 11 ^th ^March, 2020. The virus has been responsible for ’locking down’ much of the human race in most countries of the world for several months. The infectivity of this virus is extremely high and the mortality of this disease is around 5–7 percentage of the total infected cases ^1^. However, the infection fatality rate was reported to be 0.5–3.6% and the case fatality rate 2.3–7.2%, as per other reports since it differs from region to region over time ^2^

Coronaviruses consist of an enveloped RNA genome, and these viruses are zoonotic in nature. They cause respiratory, hepatic neurological, and enteric diseases in birds and mammals. As per the recent reports, this disease has claimed 962,634 lives worldwide, infecting 31,175,835 people globally till 22 ^nd ^September, 2020 ^3^. SARS-CoV-2 consists of 30 kb nucleotides as its genome ^4^. Two polyproteins essential for replication and transcription, pp1a and pp1ab, are encoded by the SARS CoV-2 replicase gene. The main protease (3C-like protease) or Mpro (a 33.8 kDa protein) is responsible for releasing the functional viral polypeptides from polyproteins after cleaving pp1a and pp1ab ^4^. Therefore, Mpro is identified as an important drug target owing to its functional significance in the life cycle of the virus and also due to the absence of its homologues in the human race ^4^.

A few drugs such as hydroxychloroquine and remdesivir that were found via bioinformatics approach have been reported. Hydroxychloroquine is a drug that is presently used against malaria. But when it was tested on SARS-CoV-2 virus, it showed activity against the virus in vitro. However, it was not found to be effective against the virus in COVID 19 patients during clinical trials ^5, 6^. Later, this drug was prohibited from use in certain countries due to it side effects. Remdesivir is an antiviral drug that was found to show activity against the virus and it did inhibit the SARS-CoV-2 virus to certain extent. Remdesivir is presently used against COVID-19 in certain countries ^7^.

In the present work, we carried out high throughput virtual screening (HTVS). It is a rapid way to search for the potential drug against the target. HTVS of Zinc natural database was performed through Python prescription software known as PyRx. This software has in built dock, Vina and Autodock tools along with openbabel. Autodock software utilizes MGLTools that has computer aided drug discovery pipeline. CADD is required for virtual high throughput screening of large databases for probable hits as therapeutic agents. This type of virtual screening helps to dock several ligands on one protein. PyRx is publicly obtainable HTVS software. Analysis and identification of distinct poses visibly and using a scoring algorithm quantitatively forms two basis of docking technique ^8^.

The free binding energy (ΔG) between the protein and the ligands is calculated in docking. The free binding energy, thus calculated, forms the foundations of complex systems of subjects such as molecular biology and biochemistry ^8^.

## Methods

### Molecule selection

As per our earlier published article ^8^, we found that fexofenadine acetate (Allegra), atovaquone, ethamidindole, baicalin,justicidin D, euphol,curine and glycyrrhetic acid could be used as COVID-19 inhibitory drugs ^8^. In that article, we selected 129 known drugs for drug repurposing, from which we found atovaquone, presently in use for treating malaria and *Pneumocystis carinii* pneumonia. We had considered another aspect in which we had taken 992 small molecules from Zinc natural database from which fexofenadine acetate (Allegra), an already in use drug for anti-allergic medicine ^8^. Notably, the small molecules from natural database had least binding energy. Therefore, we decided to dock 1100 small molecules (see *Extended data* ^9^) from the ZINC Natural Products database to identify additional lead molecules against the disease.

### Molecular docking

In this study, we utilized a bioinformatic approach that uses predictions for the arrangement of a molecule bounded to another molecule, known as molecular docking ^10, 11^. Molecular docking can be performed by two main approaches. The former describes complementation surfaces of the ligand and the protein ^12^. The latter simulates the docking process that calculates the free binding energy ΔG while protein-ligand interaction ^13– 15^. We have used PyRx (Python prescription) version 0.8 that performs HTVS. PyRx uses Autodock, ock and Vina as docking tools. Further, MGLTools, providing a computer aided drug discovery or rational drug design pipeline to search large databases to obtain potential leads as therapeutic drugs, is present as inbuilt software in the Autodock tool of PyRx. 

The X-ray crystal structure of Mpro (PDB ID: 6M03 (resolution: 2 Å) was considered for our study Å). The database considered for docking was the ZINC Natural Products database. For processing of ligands and macromolecules, the energy of 3D molecules were minimized. The Universal Force Field or UFF was used for the energy minimization of small molecules before docking. Removal of water molecules and insertion of polar hydrogen into the protein crystal structure was carried out in pre-processing of the macromolecule. Later, PDBQT files of ligands and macromolecule were generated using AutoDock version 4. 

### Molecular docking process

The grid box was as per our previous article, with dimension coordinates of X:29.9242, Y:64.1097, and Z:48.1126 and center coordinates of X:12.2632, Y:12.3998, and Z:5.4737) ^8^. Maximum grid box space was chosen for docking in order to maximize the possibility of finding a good lead molecule owing to the fact that there are still no known molecule to inhibit the virus completely. Therefore, it was thought to be better to maximize the active site space. Docking was performed with the Vina software present in PyRx tool, in which the protein-ligand interaction was studied. Analysis of separate poses were done in the AutoDock and the docked pose with the least binding affinity in kcal/mol was chosen for further analysis.

Conversion of these final docked ligands into PDB format was done using Pymol software version 1.7.4; Discovery studio 4.1 was used to study their interaction. Pymol could also be used for studying the interactions. 

In addition, remdesivir, an antiviral agent that was found to act to some extent against this disease, was used as the reference molecule. Hence, it was initially docked with 6M03 in order to know (ΔG) value as the reference range for acquiring the hits.

## Results

### Docking results

Mpro has six stranded antiparallel beta barrels which provides the substrate binding site between domain I (10–99 residues) and domain II (100–182 residues) ^16^. Dimerization of Mpro is regulated by domain III(198–303 residues) which is a globular cluster of five helices. It is primarily done by forming a salt-bridge interaction between Glu290 of one protomer and Arg4 of the other ^16^. The two molecules domain II of molecule A and the NH2-terminal residues(“N-finger”) of molecule B are arranged perpendicularly to one another to provide a tight dimer for Mpro ^16^. N-finger of each of the two protomers is found to be interacting with Glu166 of the other protomer, therefore, the N-finger is firmly pressed in between domains II and III of the parent monomer and domain II of the other monomer. Dimerization is essential for the catalytic activity of Mpro. Hence, these residues (10 to 99, 100 to 182 and 198 to 303) are essential for increasing the catalytic activity of Mpro ^16^. Docking results for each molecule are given in the *Extended data* ^9^.

### Docking results for top 25 molecules against SARS-CoV-2 Mpro

**Figure 1.**
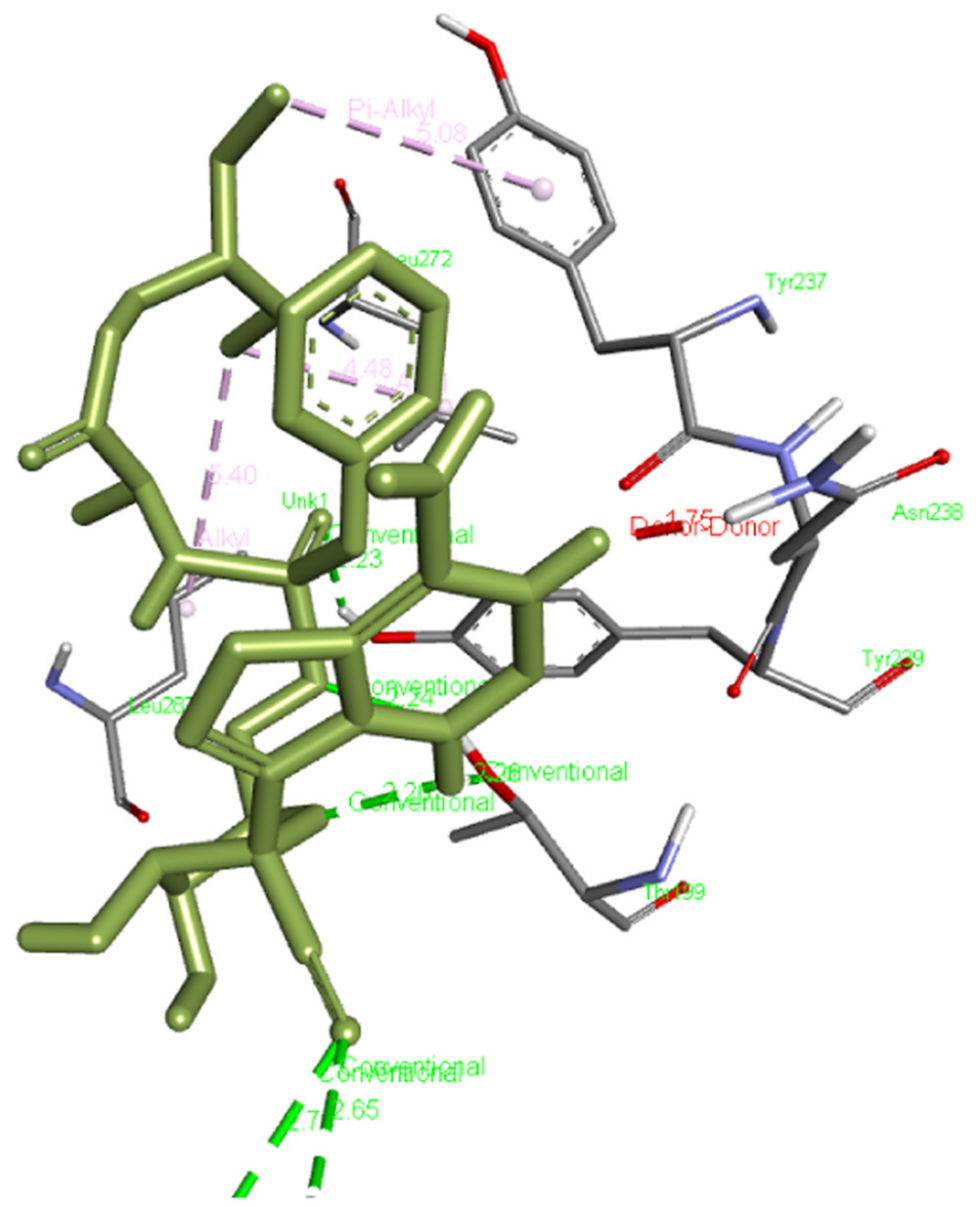
Interaction of remdesivir with Mpro.

The free binding energy for our reference molecule, which is remdesivir, was found to be -6.3 kcal/mol and it interacted with Leu272, Leu287, Tyr237, and Asn238 forming six hydrogen bonds with Lys137, Thr199, and Tyr239 residues of the main protease, Mpro (6M03). Figure 1 presents the interaction of remdesivir with Mpro.

The best 25 molecules, with binding energies of -8.8 kcal/mol to -8.7 kcal/mol, were shortlisted and are depicted in Table 1. 

**Table 1.** Docking results of top 25 hits against SARS-CoV-2 Mpro.

	Ligands Prefix *ZINC000*	Binding affinity (kcal/mol)	Molecule Formula	Common name
1	100061903	-8.8	*C*_30_ *H*_38_ *N*_2_ *O*_5_	Ramipril Benzyl Ester
2	027865962	-8.8	*C*_19_ *H*_17_ *NO*_4_	Escholtzine / (S)-Stylopine
3	001205247	-8.8	*C*_30_ *H*_33_ *NO*_3_	4’-(Decyloxy)-1,1’-biphenyl-4-carboxylic acid 4-isocyanophenyl estere
4	042835388	-8.8	*C*_35_ *H*_56_ *O*_7_	Epocholeone
5	008783205	-8.8	*C*_20_ *H*_13_ *N*_3_ *O*_7_	2,4,6-Trinitrophenol-phenanthrene(1:1)
6	004430007	-8.8	*C*_30_ *H*_44_ *O*_4_	3-Oxoglycyrrhetinic acid
7	004098157	-8.8	*C*_20_ *H*_24_ *O*_6_	Lariciresinol
8	014829676	-8.8	*C*_28_ *H*_32_ *O*_6_	Cochinchinone B
9	072320605	-8.8	*C*_26_ *H*_35_ *NO*_6_	Lactimidomycin
10	085648664	-8.8	*C*_28_ *H*_45_ *NO*_3_	5-Methoxy-1-indanone O-stearoyloxime
11	085593575	-8.8	*C*_23_ *H*_22_ *O*_5_6	Rotenone
12	085593490	-8.8	*C*_32_ *H*_32_ *O*_11_	Methyl (E)-3-[10-(4-hydroxy-3-methoxyphenyl)-9-(hydroxymethyl)-6,13-dimethoxy- 15-[(E)-3-methoxy-3-oxoprop-1-enyl]-8,11-dioxatricyclo[10.4.0.02,7] hexadeca- 1(12),2(7),3,5,13,15-hexaen-4-yl]prop-2-enoate
13	085593420	-8.8	*C*_31_ *H*_28_ *O*_11_	Cardinalin 4
14	085593150	-8.8	*C*_34_ *H*_32_ *N*_2_ *O*_9_	4-[(4-Aminophenyl)methyl]aniline;4-(4-carboxy-3 -ethoxycarbonylbenzoyl)-2- ethoxycarbonylbenzoic acid [(4S,5S,6S,7S,8S,9R)-7-Anilino-8-hydroxy-8 -(hydroxymethyl)- 9-(methoxymethoxy)-4,9-dimethyl-2 -phenyl-3-oxa-1-azaspiro[4.4]non-1-en-6-yl] 4- nitrobenzoate
15	085593179	-8.8	*C*_31_ *H*_33_ *N*_3_ *O*_9_	
16	002127691	-8.7	*C*_26_ *H*_34_ *O*_6_	Cinobufagin
17	118913911	-8.7	*C*_19_ *H*_28_ *O*_3_	4-Hydroxytestosterone
18	100061900	-8.7	*C*_30_ *H*_38_ *N*_2_ *O*_5_	6-(4-Methylpiperazin-1-yl)-6-[3-[2-(2-phenylethyl) phenoxy]propoxycarbonyl]cyclohex-3- ene-1-carboxylic acid
19	004097834	-8.7	*C*_30_ *H*_46_ *O*_5_	Quillaic Acid
20	014883275	-8.7	*C*_27_ *H*_24_ *O*_5_	Vitisinol C
21	015119492	-8.7	*C*_20_ *H*_16_ *O*_6_	Citrusinol
22	072320614	-8.7	*C*_31_ *H*_43_ *N*_7_ *O*_6_	Segetalin A
23	085648346	-8.7	*C*_39_ *H*_45_ *NO*_6_	Propafenone dimer
24	085628204	-8.7	*C*_33_ *H*_54_ *N*_4_ *O*_5_	2-[2-Methylpropanoyl(octadecyl)amino]-4-oxo-4-[(3-oxo-1,2-dihydroindazol-6- yl)amino]butanoic acid
25	085593555	-8.7	*C*_24_ *H*_25_ *NO*_6_	Scopolamine 4-hydroxybenzoate

The results obtained through the molecular docking analysis of some of the presented molecules exhibit different insights that allows asserting whether it is possible to use them for medical treatment of COVID-19.

**Figure 2.**
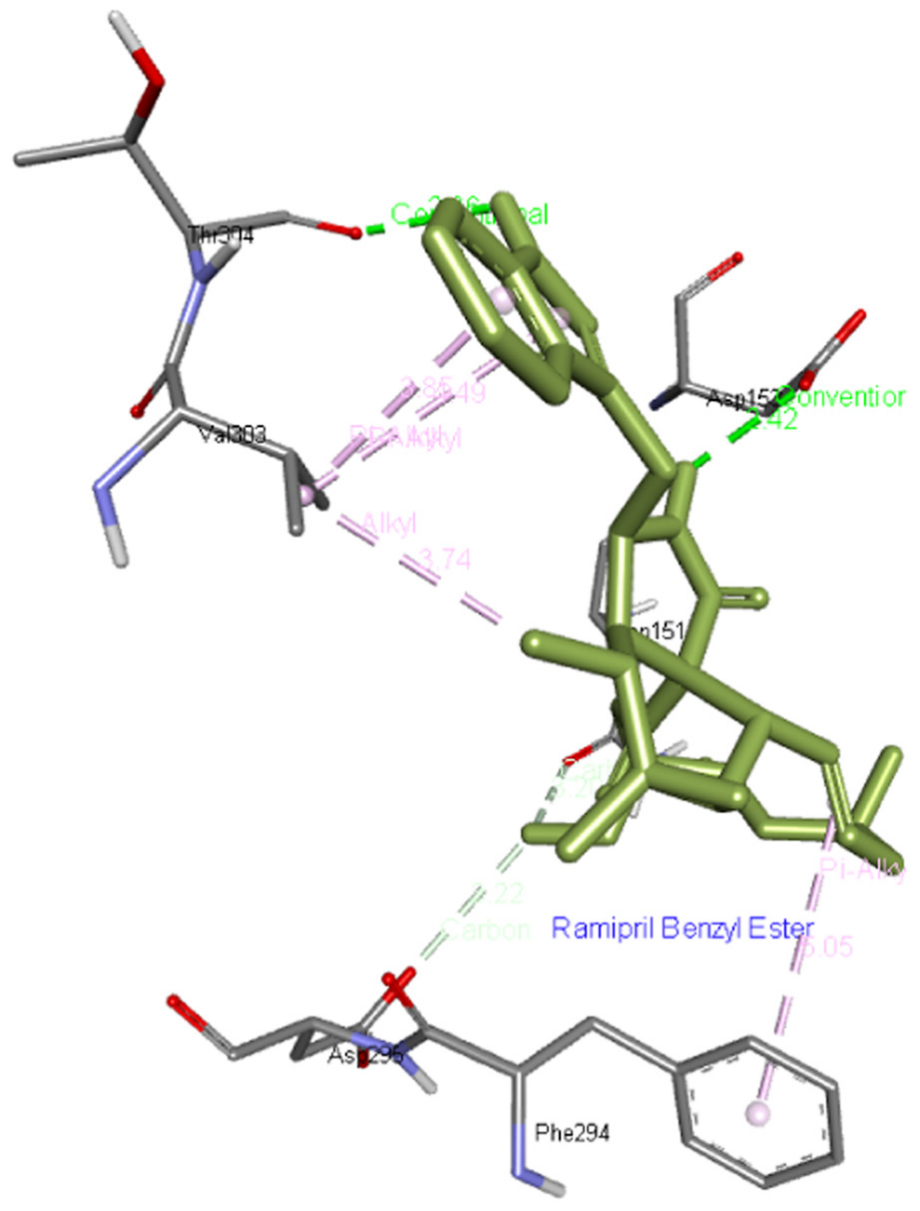
Interaction of ramipril benzyl ester with Mpro.

**Figure 3.**
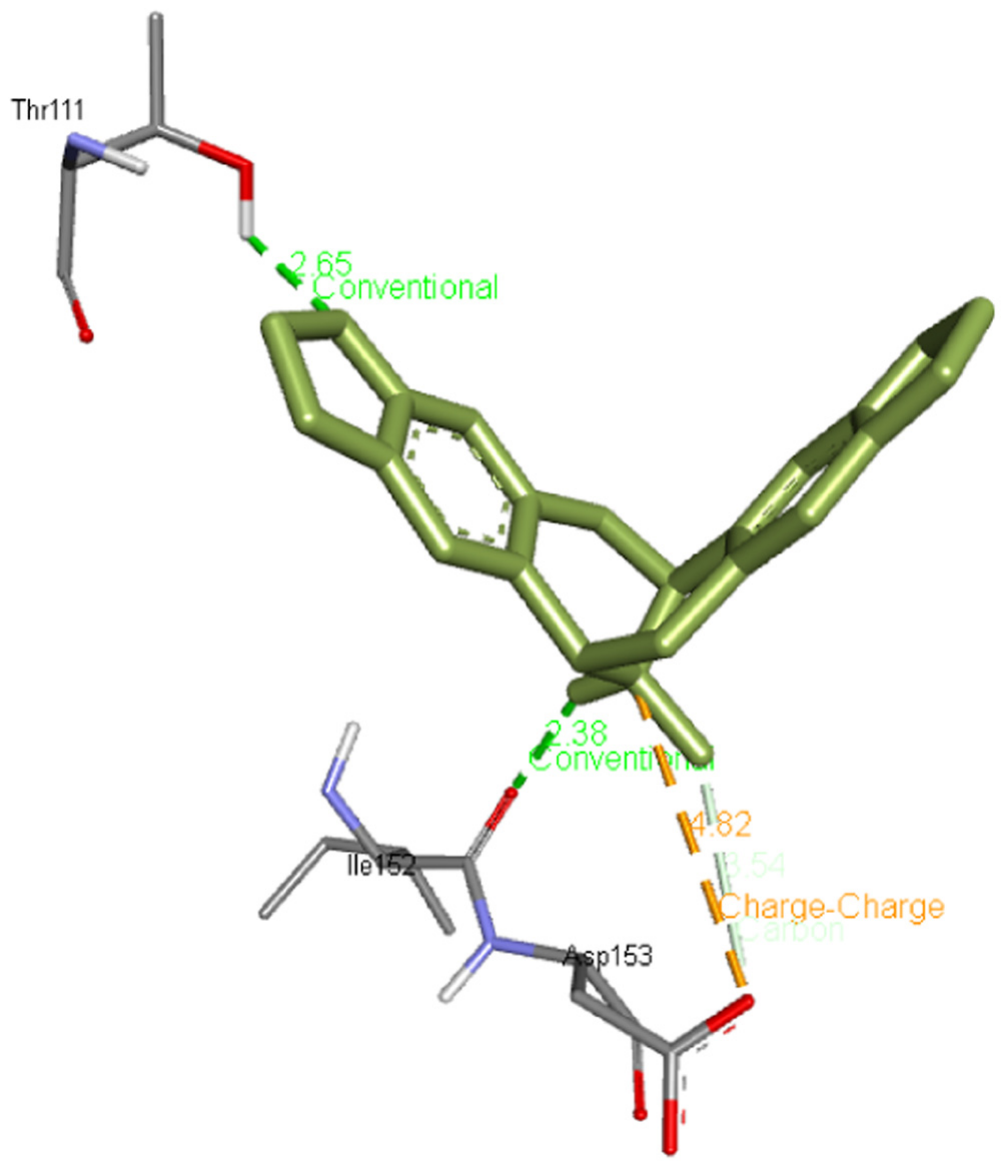
Interaction of escholtzine / (S)-stylopine with Mpro.

**Figure 4.**
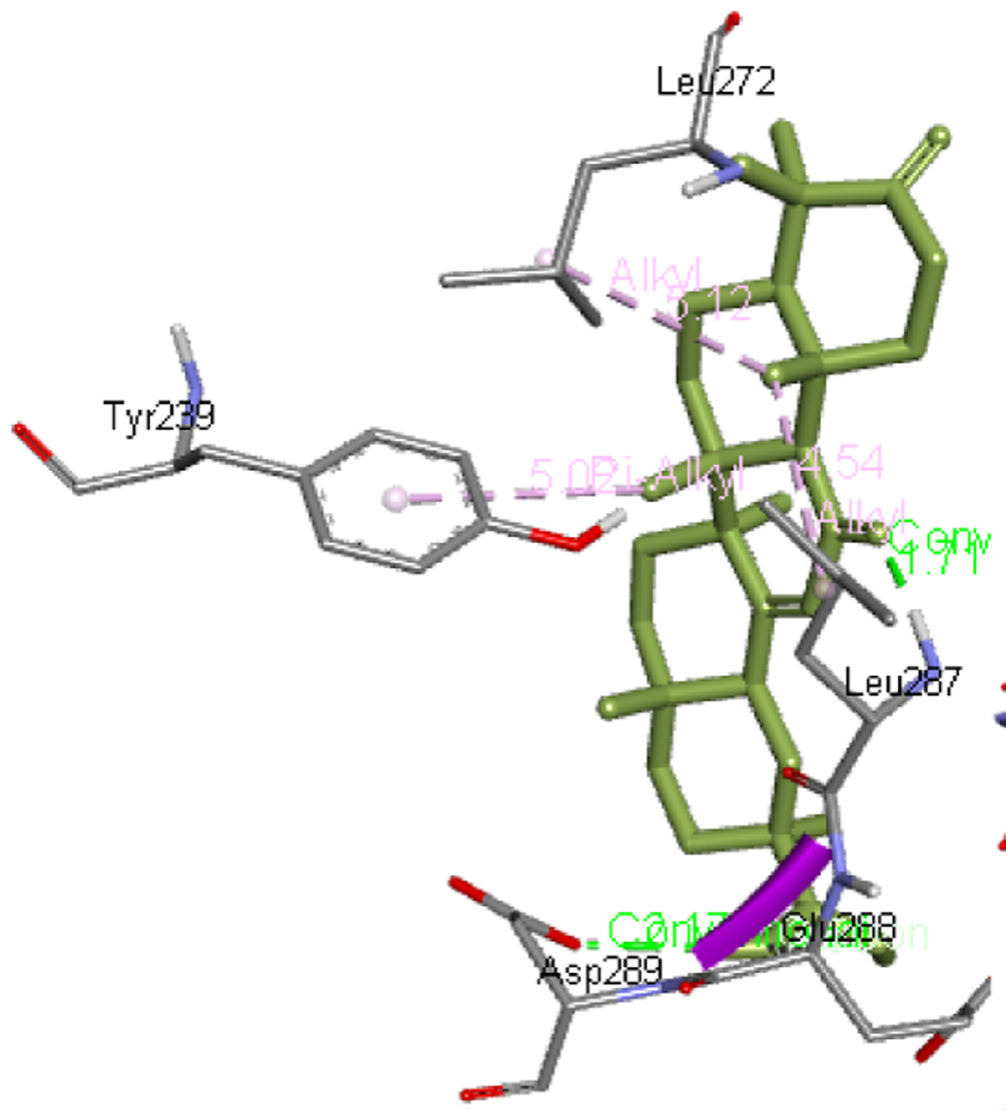
Interaction of 3-oxoglycyrrhetinic acid with Mpro.

**Figure 5.**
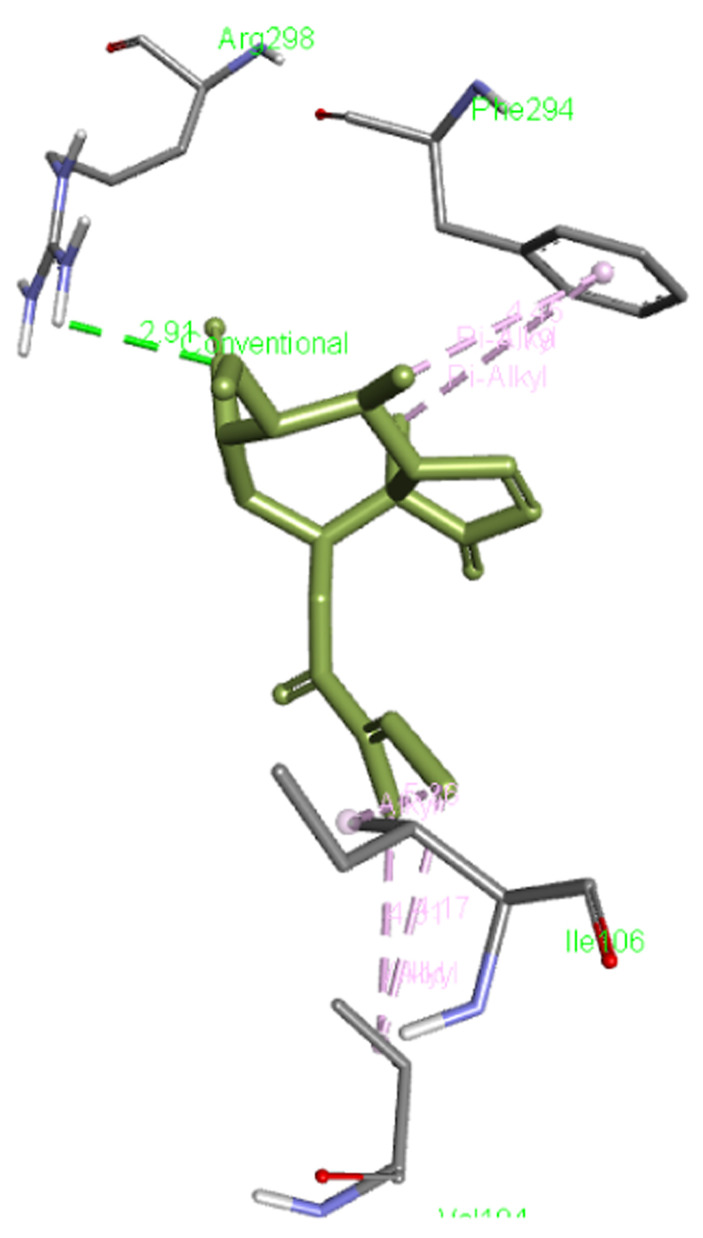
Interaction of lariciresinol with Mpro.

**Figure 6.**
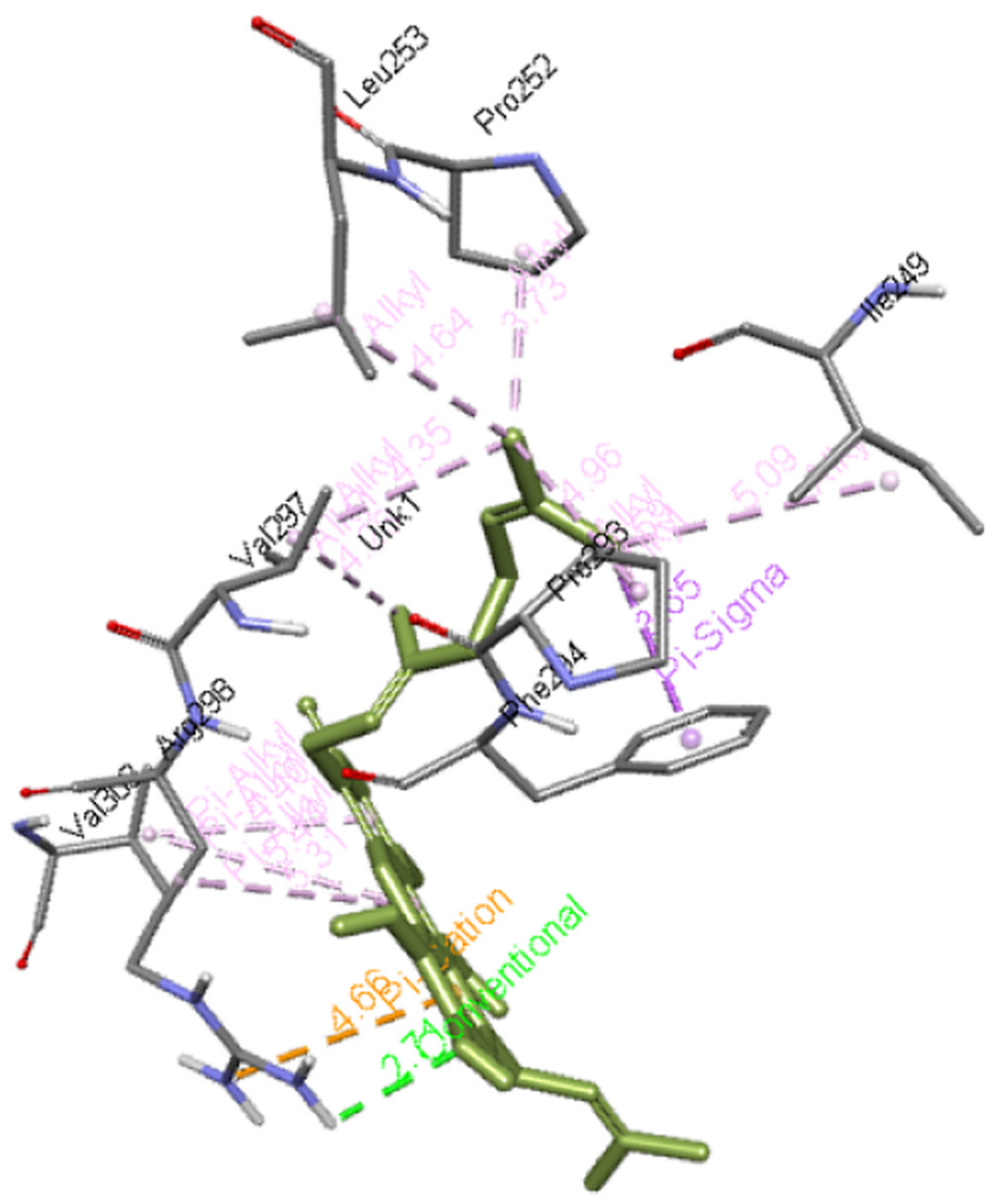
Interaction of cochinchinone B with Mpro.

**Figure 7.**
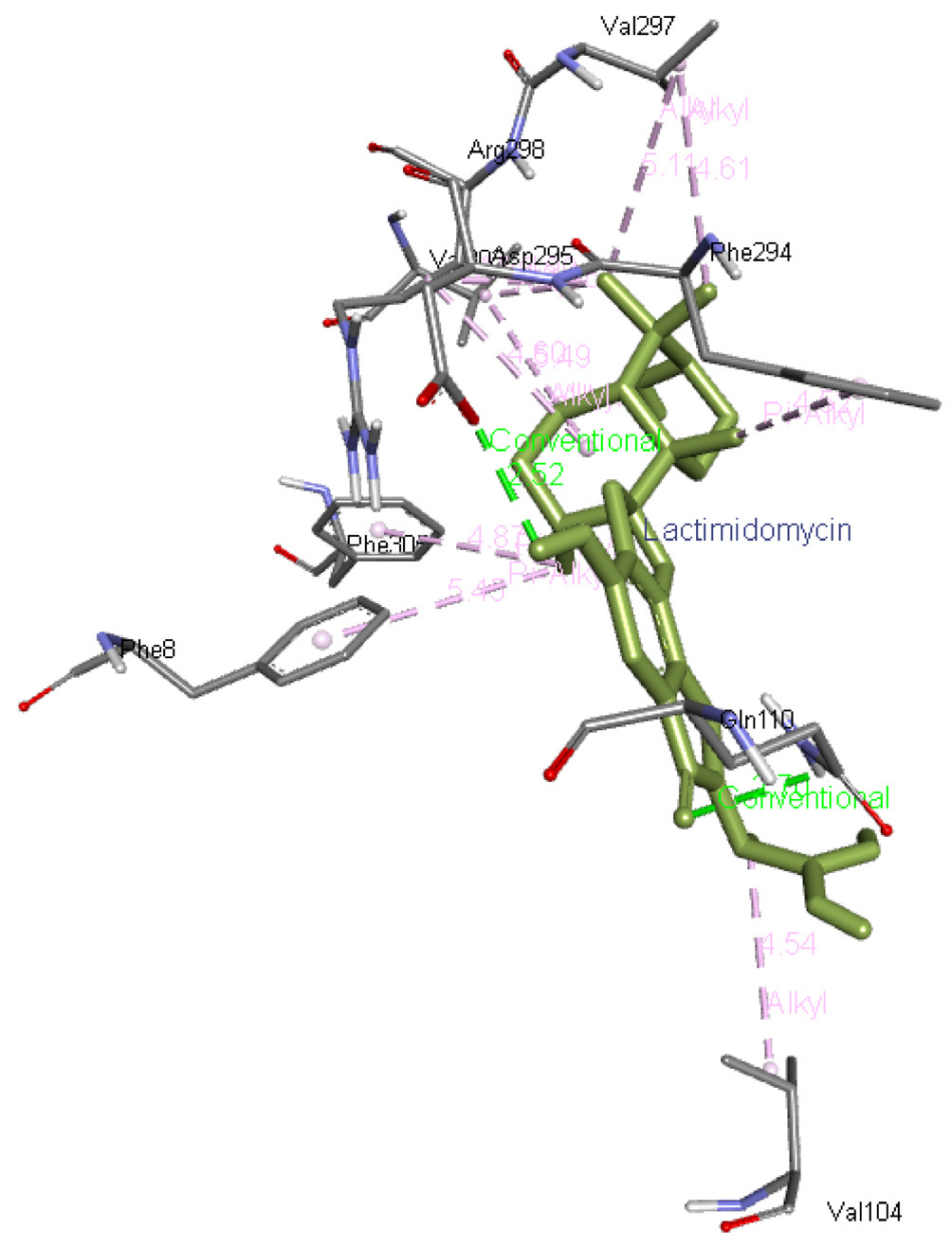
Interaction of lactimidomycin with Mpro.

**Figure 8.**
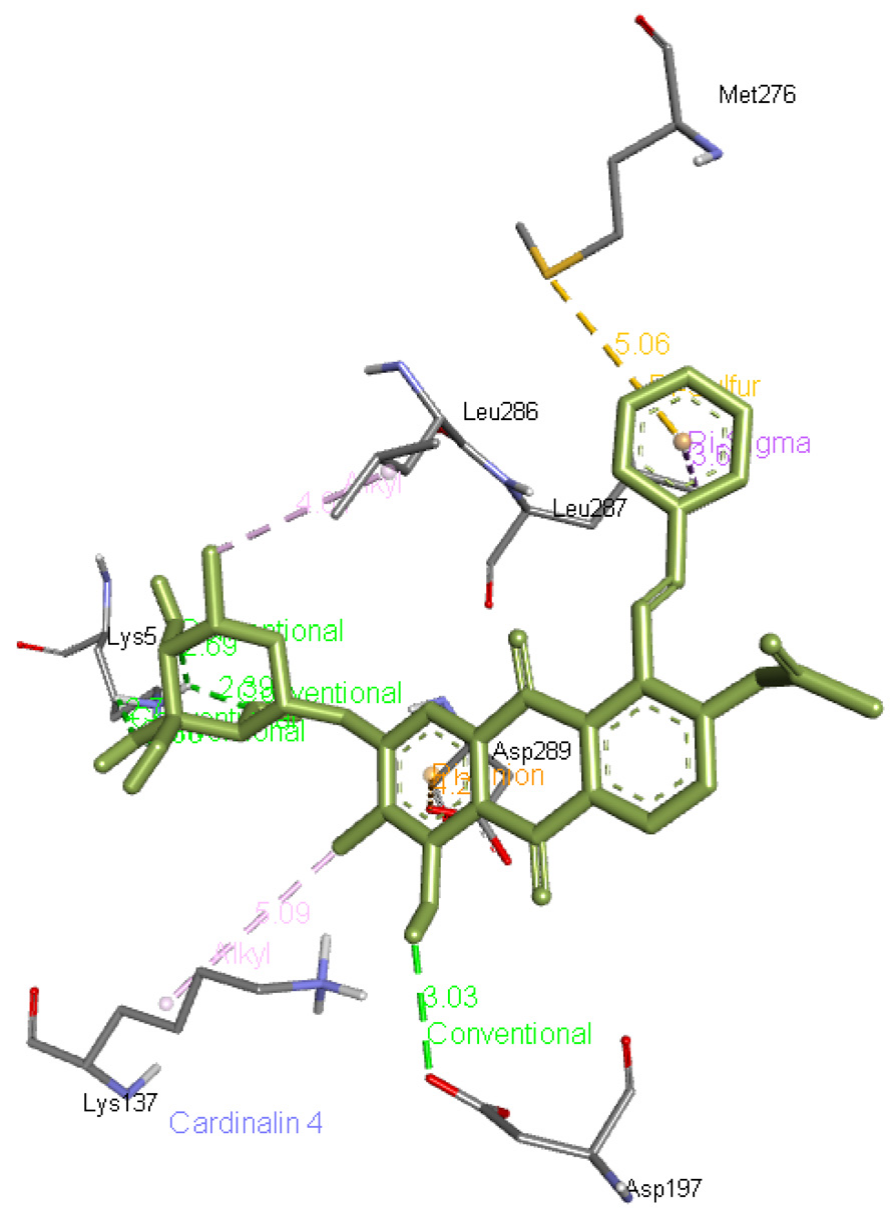
Interaction of cardinalin 4 with 6M03.

**Figure 9.**
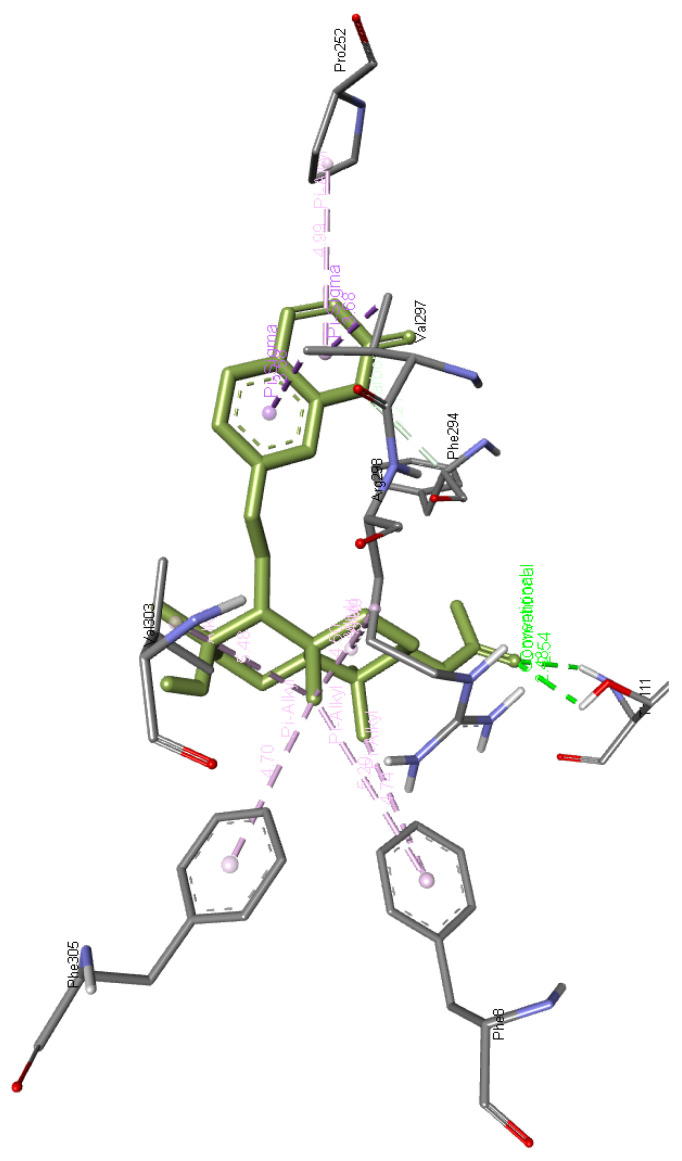
Interaction of cinobufagin with Mpro.

**Figure 10.**
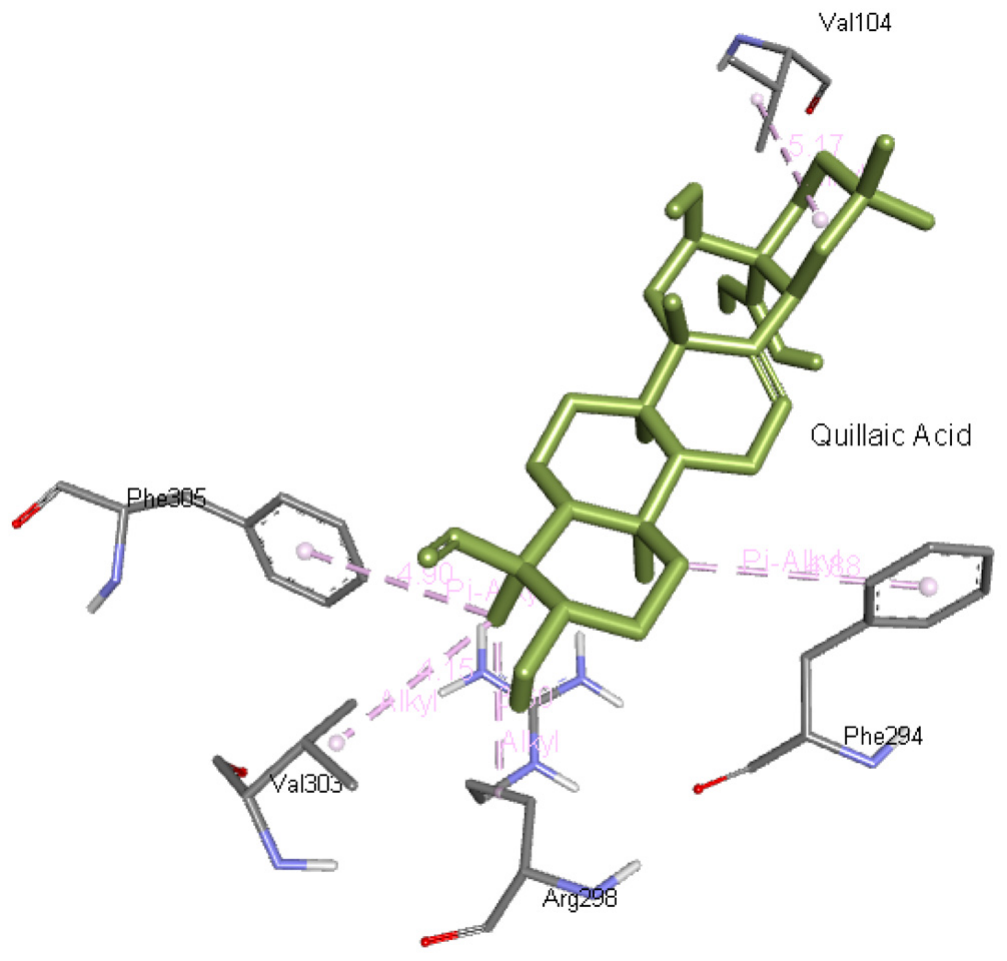
Interaction of quillaic acid with Mpro.

**Figure 11.**
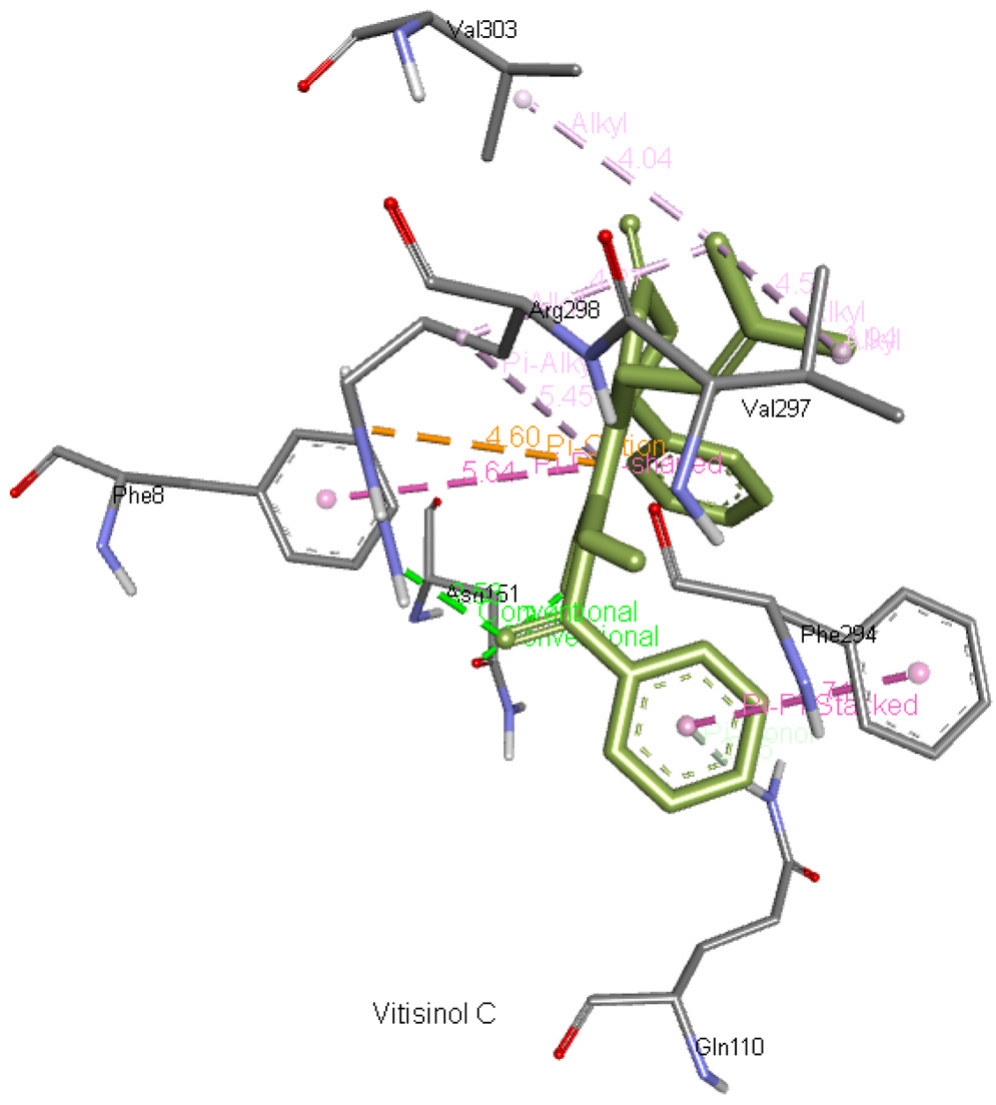
Interaction of vitisinol C with 6M03.

**Figure 12.**
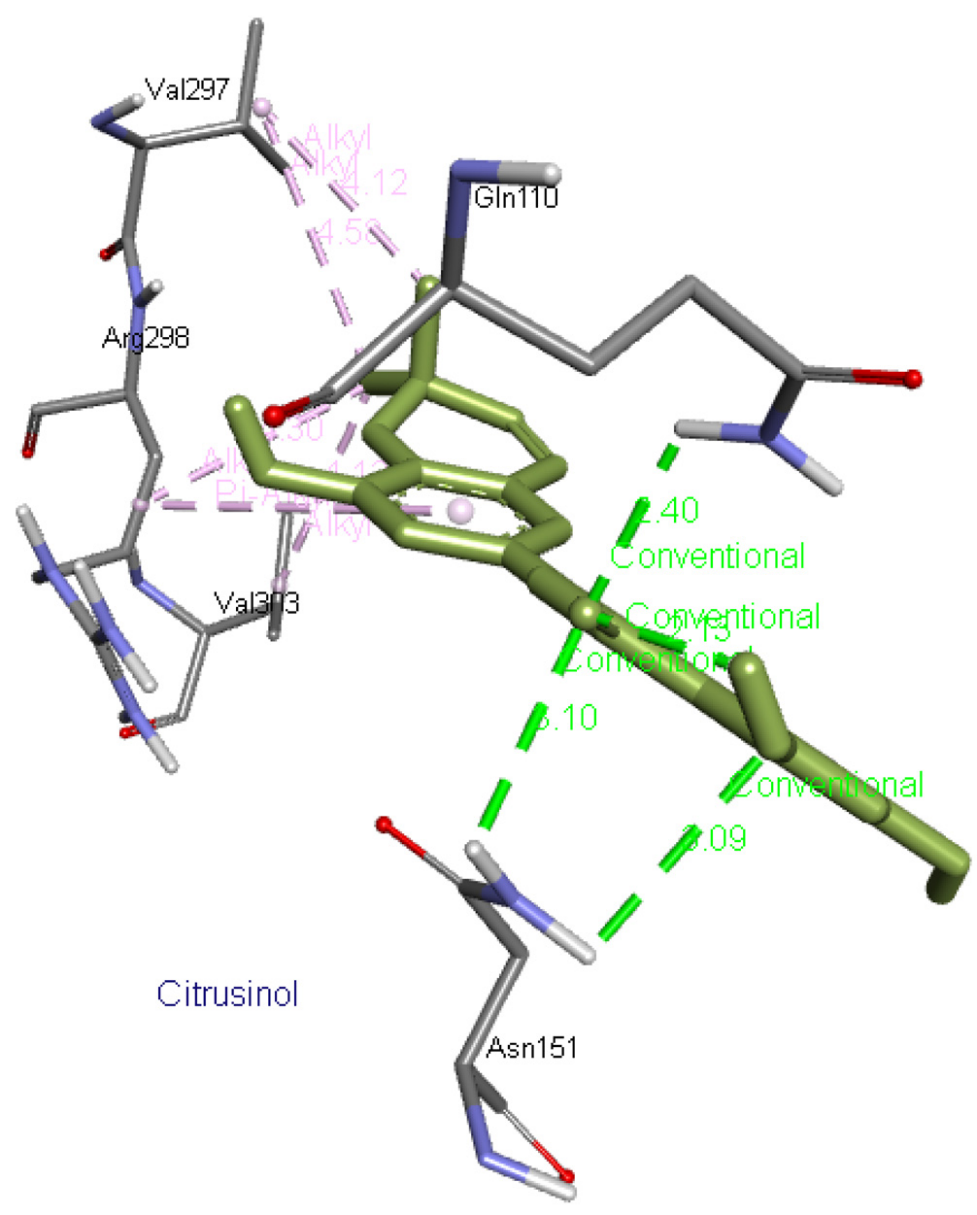
Interaction of citrusinol with 6M03.

**Figure 13.**
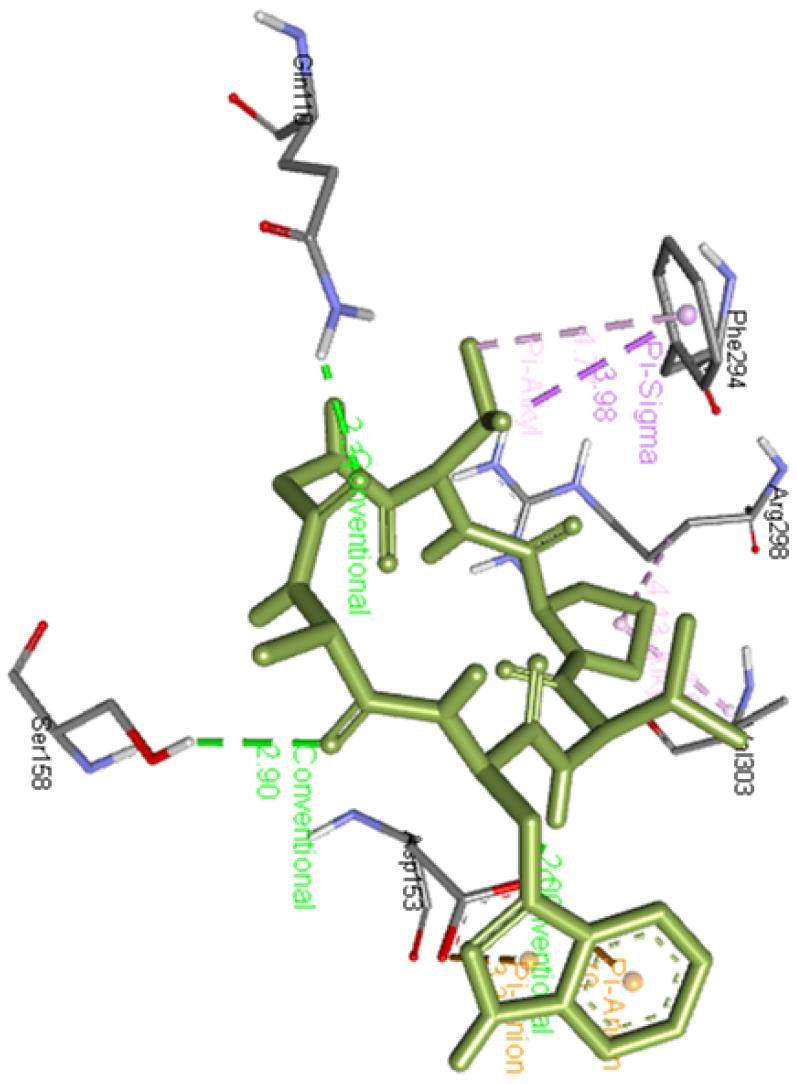
Interaction of segetalin A with Mpro.

**Figure 14.**
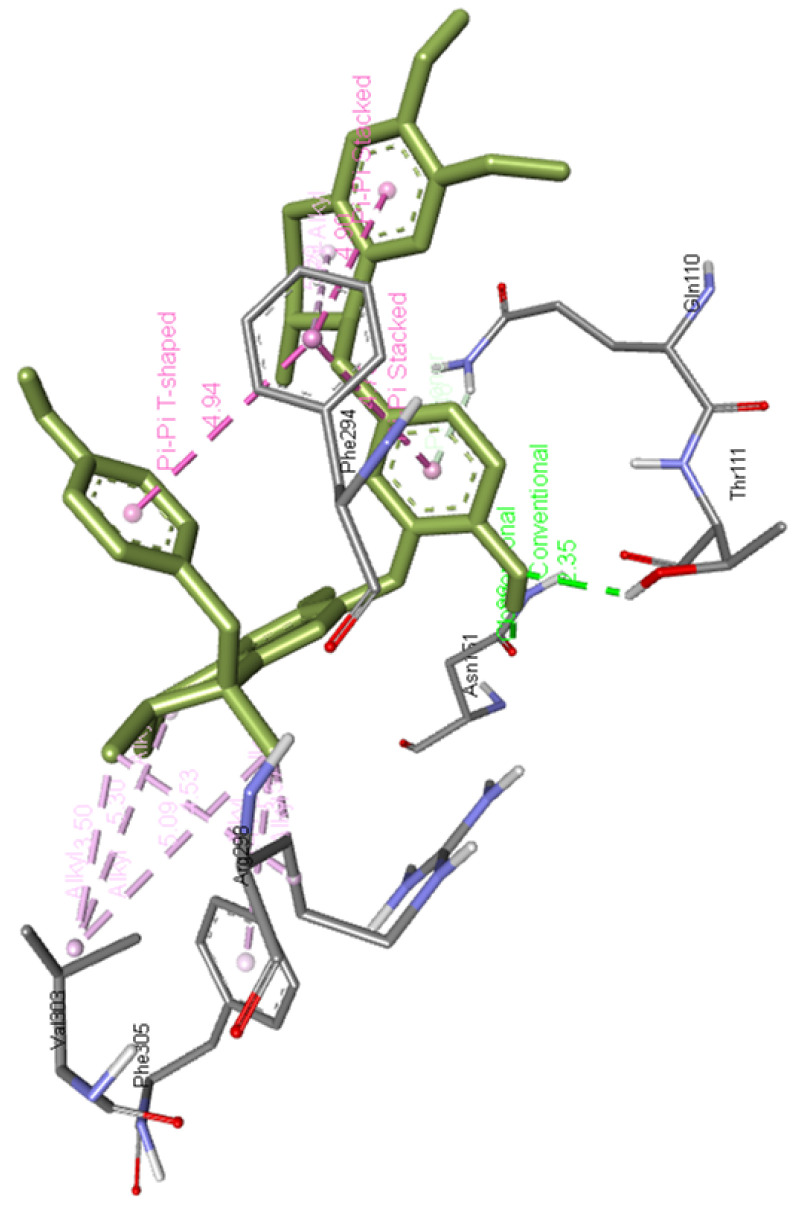
Interaction of propafenone dimer with Mpro.

**Figure 15.**
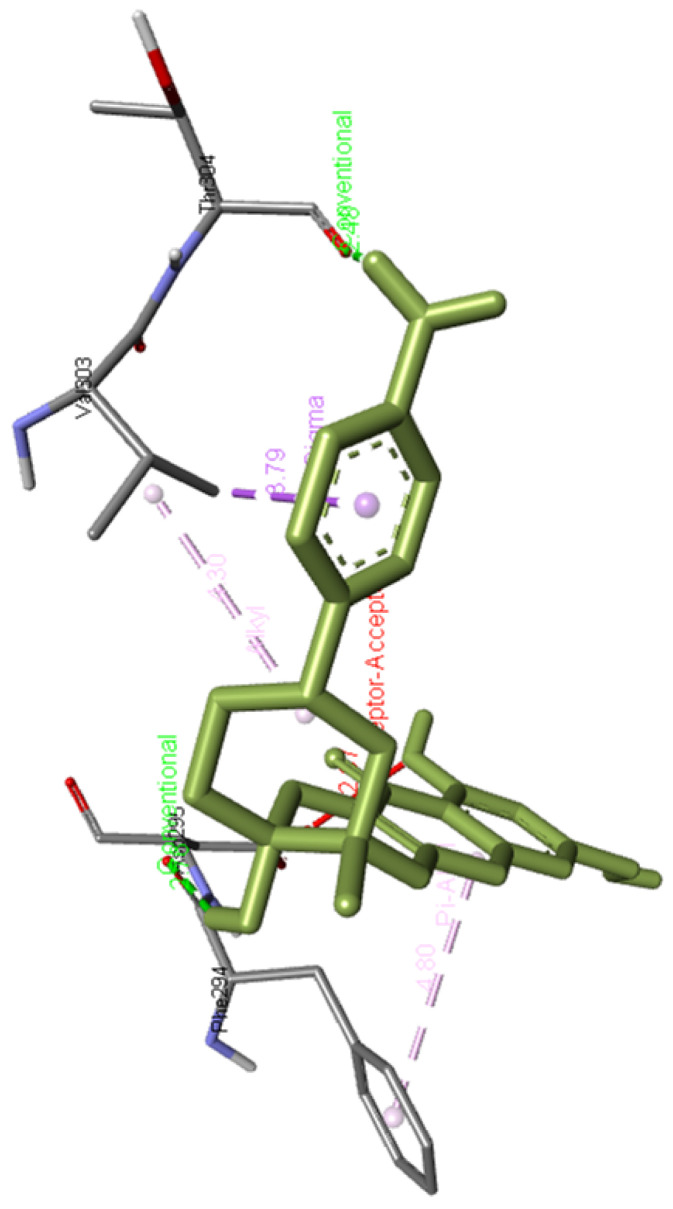
Interaction of scopolamine 4-hydroxybenzoate with Mpro.

- **Ramipril benzyl ester** forms two hydrogen bonds with Asp153 as well as Thr304 residues apart from interacting with Asn151, Phe294, Asp295, and Val303 residues of Mpro as presented in Figure 2.
- **Escholtzine / (S)-stylopine** forms two hydrogen bonds with Thr111 and Ile152 apart from interacting with Asp153 residues of Mpro. Its interaction is presented in Figure 3.
- **3-oxoglycyrrhetinic acid** forms two hydrogen bonds with Leu287 as well as Asp289, whereas Glu288, Leu272, and Tyr239 are the other interacting residues of Mpro. Figure 4 presents 3-oxoglycyrrhetinic acid interaction with Mpro.
- **Lariciresinol** forms a hydrogen bond with Arg298 as well as interacts with Phe294, Ile106, and Val104 residues of Mpro. Figure 5 presents the interaction of this molecule with 6M03.
- **Cochinchinone B** forms a hydrogen bond with Arg298 along with interacting with Ile249, Pro252, Leu253, Pro293, Phe294, Val297, and Val303 residues of Mpro. The interaction of this molecule with Mpro is presented in Figure 6
- **Lactimidomycin** interacts with Phe8, Phe305, Val303, Arg298, Phe294, Val297, and Val104. In addition, it forms two hydrogen bonds with Gln110 and Asp295 residues of Mpro as presented in Figure 7.
- **Cardinalin 4** forms five hydrogen bonds, one with Asp197 and four with Lys5 apart from interacting with other residues of Mpro such as Lys137, Met276, Leu286, Leu287, and Asp289. Its interaction with Mpro is presented in Figure 8
- **Cinobufagin** forms two hydrogen bonds with Thr111 residue and interacts with Val297, Phe294, Arg298, Val303, Phe305, Phe8, and Pro252 residues of Mpro. Figure 9 presents Ccnobufagin interaction with Mpro.
- **Quillaic acid** interacts with Val104, Phe294, Arg298, Val303, and Phe305 residues of 6M03. The quillaic acid interaction with Mpro is presented in Figure 10.
- **Vitisinol C** forms two hydrogen bonds with Asn151 and Arg298 apart from interacting with Gln110, Phe294, Val297, Val303, and Phe8. Figure 11 presents vitisinol C interaction with Mpro.
- **Citrusinol** forms four hydrogen bonds with two residues, Asn151 and Gln110, as well as interacts with Val297, Arg298, and Val303 residues of Mpro. Figure 12 presents its interaction with Mpro.
- **Segetalin A** forms three hydrogen bonds with Gln110, Ser158, and Asp153 and interacting with residues such as Phe294, Arg298, Val303 of Mpro. Figure 13 presents segetalin A interaction with 6M03.
- **Propafenone dimer** interacts with Gln110, Phe294, Arg298, Val303, and Phe305 and forms two hydrogen bonds with Thr111 and Asn151 of Mpro residues as presented in Figure 14
- **Scopolamine 4-hydroxybenzoate** forms hydrogen bonds with Phe294 and Thr304 apart from interacting with Asp295 and Val303 residues of Mpro. Figure 15 presents the scopolamine 4-hydroxybenzoate interaction.

## Discussion

Natural molecules are known derivatives of most of the present drugs in use against many diseases. In this study, we have also found some good leads and most of the 25 selected molecules show medicinal properties. Ramipril benzyl ester (protected form of ramipril), which is an inhibitor of angiotensin-converting enzyme ACE is the host receptor protein where the SARS-CoV-2 spike protein binds. Ramiprilat, which is an active compound of ramipril benzyl ester, is formed on the hydrolysis of the ester group, which later inhibits ACE. This leads to the prevention of the catalytic conversion of angiotensin I to angiotensin II that causes vasodilation ^17^. Stylopine ^18^ and quillaic acid exhibits anti-inflammatory activity ^19^. Cinobufagin, CBF, has antitumor activity as it is known to inhibit the growth of PC3 cells both *in vitro* and *in vivo* along with inducing apoptosis of tumor cells as per reports ^20^. It was found that intake of the plant ligands such as lariciresinol reduced endothelial dysfunction and vascular inflammation in post-menopausal women and middle aged/elderly men ^21^. Vitisinol C as per earlier reports was found to exhibit an essential activity against amyloid aggregation, which is present in *Vitis vinifera* grapevine shoots ^22^.

Segetalins are known to have vasorelaxant activity ^23^. Scopolamine is known as antimuscarinics that blocks the effects of certain natural substance (acetylcholine) on the central nervous system, it is also used to prevent nausea and vomiting caused by motion sickness or medications used during surgery ^24^.

**Figure 16.**
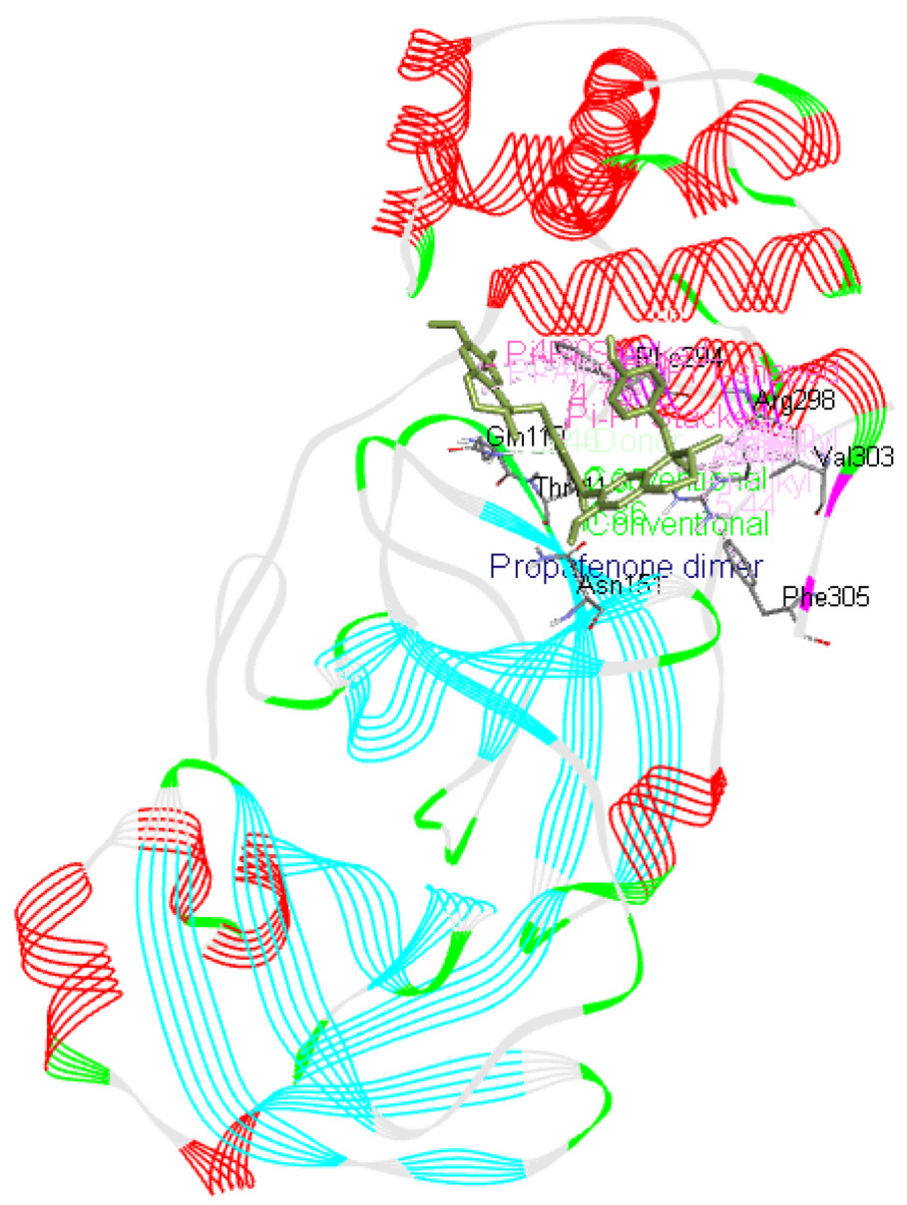
Propafenone dimer interaction image with whole Mpro, main protease(6M03) of SARS-CoV-2.

**Figure 17.**
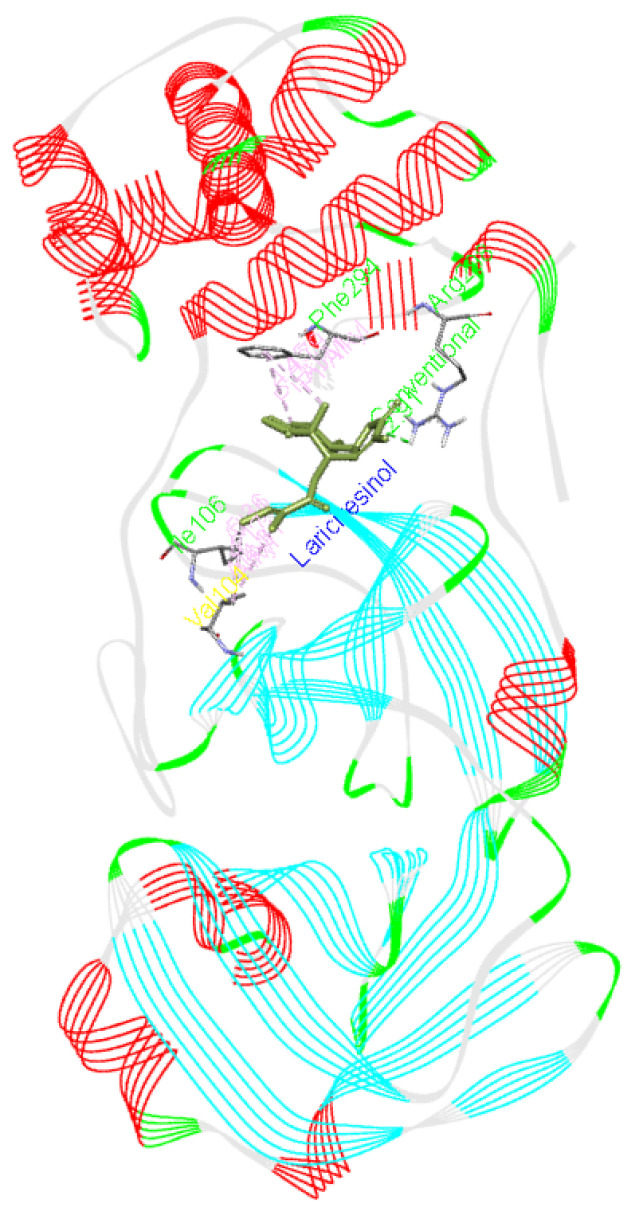
Lariciresinol interaction image with whole Mpro, main protease(6M03) of SARS-CoV-2.

**Figure 18.**
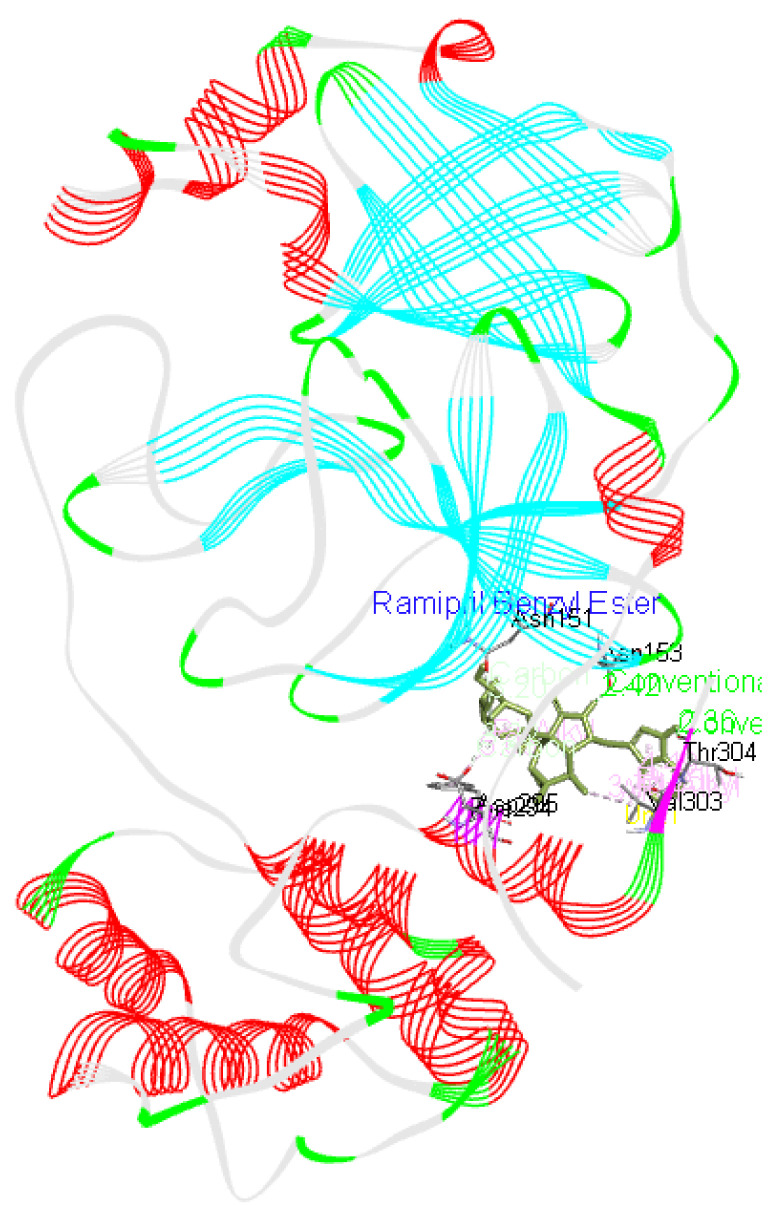
Ramipril Benzyl Ester interaction image with whole Mpro, main protease(6M03) of SARS-CoV-2.

3-oxo-glycyrrhetinic acid has antitumor activity and expectorant (antitussive) properties ^25^. Lactimidomycin has antifungal, antiviral, and anti-cancer properties apart from acting as a direct inhibitor of protein translation inribosomes which is a glutarimide antibiotic derived from the bacterium *Streptomyces amphibiosporus* ^26^. Cardinalins dinalins 4 exhibit anti-cancer properties. Conchinone B has an antioxidant activity. Rotenone used to treat head lice on humans, scabies, and parasitic mites on chickens, livestock, and pet animals. Propafenone is used for the treatment of irregular heartbeat like atrial fibrillation, paroxysmal supraventricular and tachycardia in order to restore normal heart rhythm for maintaining a regular, steady heartbeat ^27^. These small molecules or potential drugs could be used as therapeutics for COVID-19 disease due to their binding affinity with the viral protein as well as interaction with Mpro. These small molecules were found to interact with the residues involved in enhancing the catalytic activity of Mpro or SARS CoV-2 main protease. Therefore, these could prove to play important role in inhibiting the viral activity. However, there are three small molecules ramipril benzyl ester, propafenone dimer and lariciresinol found from this study that might prove to be useful against this virus especially due to their present medical application, docking score and interaction with Mpro, as illustrated in Figure 16, Figure 17 and Figure 18.

## Conclusions

Ramipril benzyl ester, stylopine, quillaic acid, cinobufagin, vitisinol C, segetalin A, scopolamine, 3-oxo glycyrrhetinic acid, lariciresinol, conchinone B, lactimidomycin, cardinalins 4, and propafenone dimer are 13 molecules that are predicted to form good interaction with Mpro. These molecules showed better binding affinity for Mpro than the reference molecule. Ramipril benzyl ester, apart from binding to the main protease of the virus, was also found to inhibit the virus receptor ACE present in humans. ACE2 is the receptor to which virus spike protein is known to bind to the host. The spike protein and Mpro of the SARS-CoV-2 virus are responsible for the virulence of the virus. Ramipril benzyl ester drug is reported to inhibit ACE and also found to inhibit main protease of SARS-CoV-2 virus from this study. Ramipril benzyl ester is presently used against heart stroke. Ramipril benzyl ester and propafenone are two important small molecules found from our study due to their present medicinal use as drugs for other diseases in human along with lariciresinol due to its medical application as stated in the discussion. As a result, these molecules have medicinal properties which could be used for the treatment of COVID-19 disease after further evaluation. This study presents a significant possibility in finding the cure for the COVID-19 disease from the drugs repurposing point of view. The drug targets mentioned in our study could bring relief to all those who are infected with the disease and these may also act as a prophylactic treatment when tested by the frontline healthcare providers. This study could provide relief to everyone if the drugs mentioned in our study proved to inhibit the virus in COVID19 positive patients since they are known natural derivatives with minimal side effects in individuals. Thus, our study could help in flattening the curve with speedy recovery in COVID -19 positive patients.

## Data availability

### Source data

The COVID-19 main protease structure was downloaded from the Protein Data Bank, ID 6M03: https://www.rcsb.org/structure/6M03. 

Ligands were obtained from the Zinc Natural Products database ( ). 

### Extended data

Zenodo: Molecular Docking ZINC DB. https://doi.org/10.5281/zenodo.4050576 ^9^. 

This project contains the following extended data:

- Docking results for thee 100 best molecules (XLSX).
- Interaction_images_viral_protein_molecules (subfolder). (Interaction images of the best 42 molecules.)
- Zinc_natural_database_pdbqt_minimized (subfolder). (Molecular docking results for 1100 small molecules from the ZINC Natural Products database.)

Extended data are available under the terms of the Creative Commons Attribution 4.0 International license (CCBY 4.0).
