# Supplementary material for: Bioinformatic study to discover natural molecules with activity against COVID-19
Source: F1000Res. 2020 Oct 6;9:1203. [Version 1] doi: 10.12688/f1000research.26731.1 (PMC7590895; doi:10.12688/f1000research.26731.1)
Supplement: Peer review history

# Peer review: Reviewer response for version 1

**DOI:** 10.5256/f1000research.29515.r72544

**Published:** 2020-10-23

**Authors:** Maria Florencia Pollo-Cattaneo

**Affiliations:** 1 Information System Methodologies Research Group GEMIS, Universidad Tecnológica Nacional Facultad Regional Buenos Aires, Buenos Aires, Argentina

The article proposes a study for the search of drugs to combat COVID-19. This search will be carried out by virtually screened the Zinc natural database using HTVS tool through molecular docking studies to analyze molecules recommended for the treatment of COVID-19.

 The methodology used is consistent with the objective and the results are promising.

 Researchers are encouraged to continue with the proposed line and advance their research.

Is the work clearly and accurately presented and does it cite the current literature?

Yes

If applicable, is the statistical analysis and its interpretation appropriate?

Yes

Are all the source data underlying the results available to ensure full reproducibility?

Yes

Is the study design appropriate and is the work technically sound?

Yes

Are the conclusions drawn adequately supported by the results?

Yes

Are sufficient details of methods and analysis provided to allow replication by others?

Yes

Reviewer Expertise:

Artificial IntelligenceIntelligent Systems

I confirm that I have read this submission and believe that I have an appropriate level of expertise to confirm that it is of an acceptable scientific standard.

# Peer review: Reviewer response for version 1

**DOI:** 10.5256/f1000research.29515.r72545

**Published:** 2020-10-12

**Authors:** Pritha Ghosh

**Affiliations:** 1 nference Labs, Bangalore, India

The manuscript by Singh and Florez presents the discovery of drugs against COVID19 using molecular docking methods.

**Major:**

1. It might be worthwhile to re-check the binding affinity values for the top hits because all of the top 15 hits have a value of -8.8 kcal/mol. Does this mean that these drugs are equally effective and can be used interchangeably?
2. The authors have also not discussed the importance of the residues Tyr237, Leu272, Leu287 etc. Are these known to be critical residues in the functioning of the main protease?

**Minor:**

1. Please use a uniform nomenclature (COVID 19 or COVID-19).
2. The manuscript needs to be proofread by a proficient English speaker.

Is the work clearly and accurately presented and does it cite the current literature?

Yes

If applicable, is the statistical analysis and its interpretation appropriate?

Not applicable

Are all the source data underlying the results available to ensure full reproducibility?

Yes

Is the study design appropriate and is the work technically sound?

Yes

Are the conclusions drawn adequately supported by the results?

Yes

Are sufficient details of methods and analysis provided to allow replication by others?

Yes

Reviewer Expertise:

Bioinformatics

I confirm that I have read this submission and believe that I have an appropriate level of expertise to confirm that it is of an acceptable scientific standard.
